# Delayed-Onset Post-Stroke Delusional Disorder: A Case Report

**DOI:** 10.3233/BEN-120315

**Published:** 2013

**Authors:** Raíssa B. Barboza, Gabriel R. De Freitas, Fernanda Tovar-Moll, Leonardo F. Fontenelle

**Affiliations:** ^1^Departamento de Psiquiatria e Medicina Legal and Instituto de Psiquiatria, Programa de Ansiedade e Depressão, Universidade Federal do Rio de Janeiro, Rio de Janeiro, RJBrazil; ^2^Departamento de Psiquiatria e Saúde Mental, Universidade Federal Fluminense, Niterói, RJBrazil; ^3^Instituto D’Or de Pesquisa e Ensino (IDOR), Rio de Janeiro, RJBrazil; ^4^Serviço de Neurologia, Universidade Federal Fluminense, Niterçi, RJBrazil; ^5^Instituto de Ciências Biomédicas, Universidade Federal do Rio de Janeiro, Rio de Janeiro, RJBrazil

**Keywords:** Stroke, psychotic symptoms, persistent delusional disorder

## Abstract

Although the prevalence of neuropsychiatric disorders among patients with cerebrovascular illness is relatively high, there are only few case reports describing post-stroke psychotic symptoms. In general, post-stroke psychoses have been reported to emerge few days after the vascular event and to vanish soon afterwards. In this report, we describe delayed-onset post-stroke delusional disorder, persecutory type. A middle-aged female patient developed a persistent delusional disorder with homicidal behavior about one year after a cerebrovascular accident affecting the right fronto-temporo-parietal region and a long period of chronic post-stroke mixed anxiety and depressive symptoms. Our case suggests that there might be long intervals between stroke and the appearance of psychotic symptoms.

## 1. Introduction

Cerebrovascular diseases are the first cause of death in Brazil and the second leading cause of death worldwide [1,2]. In Brazil, from every 100,000 women, 85 die as a consequence of stroke, a rate that is significantly high as compared to other countries in the American continent [1,2]. However, cerebrovascular diseases are more disabling than lethal, with at least 30% of the survivors making a incomplete recovery and a further 20% requiring assistance for activities of daily living [3]. Neuropsychiatric disorders affect 20% to 60% of stroke survivors and includes depressive (25–50%), anxiety (25–50%) and cognitive disorders (21.8–65%) [4,5], among other conditions.

Although it is known that focal lesions can lead to changes in mood and cognition [6], there are also few case reports in the literature describing stroke-related psychotic symptoms. In most of them, psychosis appeared within the first week after the vascular event [7], and usually persisted for a short period of time (i.e. up to 4 months) [8,9]. In this study, our aim is to describe the case of a patient who developed, about one year after a stroke, a persistent delusional disorder.

## 2. Case report

Ms. A, a 54 year-old, single Caucasian woman with some high school education, sought medical treatment in May 2008, complaining of headache and left leg weakness. She had a history of hypertension, dyslipidemia and glucose intolerance and reported a fainting episode with crooked mouth and slurred speech 15 days before. Ms. A also complained of sadness,

ISSN 0953-4180/13/$27.50 © 2013 – IOS Press and the authors. All rights reserved

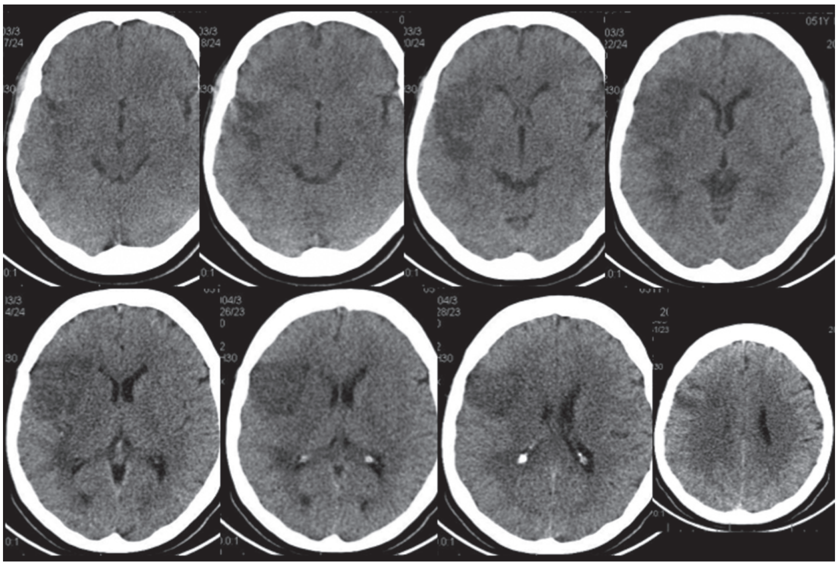

Fig. 1. Computed Tomography scan showing an acute ischemic stroke lesion in the territory of the right middle cerebral artery, extending from the right insula (top left) to the inferior and middle frontal gyri (bottom right).

hopelessness and tearfulness that began after this episode. Neurological examination showed left-sided paresis, hypoesthesia, exaggerated reflexes, and deviation of the angle of the mouth to the right. The cranial CT scan identified an acute ischemic lesion in the territory of the right middle cerebral artery, extending from the right insula to the inferior and middle frontal gyri (Fig. 1). Also, electrocardiography showed atrial fibrillation and diffuse changes in ventricular repolarization.

At first, the predominant psychiatric symptoms reported by the patient and her relatives included sadness, fear of being alone, insomnia, restlessness, hypervigilance and subclinical compulsive symptoms (i.e. she had to discard garbage and wash hands several times a day), which led to the hypothesis of an adjustment disorder. Mr. A was started on citalopram, 20 mg/day, which proved to be effective and well tolerated for almost a year. According to relatives, the patient had a pre-morbid borderline intellectual functioning. Mini-mental state examination showed a score of 24/30.

In subsequent assessments, almost a year after Ms. A's first contact with the psychiatry clinic, her relatives reported that she was having severe mood swings, irritability, verbal aggression, and delusional ideas with paranoid content, especially in relation to family members. On close examination, she had a puzzled look and odd speech. Ms. A said that people were getting into her house and subtracting her belongings, and that her relatives were trying to steal her money. She denied any auditory-verbal hallucinations. These symptoms had never been previously observed. In fact, the patient had a good social/interpersonal functioning and worked as an elderly caregiver before the stroke.

We have assessed Mrs. A with the Mini International Neuropsychiatric Interview 5.0, which was able to confirm the presence of a psychotic syndrome and to exclude mood disorders with psychotic features. According to DSM-IV-TR criteria, Mrs. A was diagnosed with Psychotic Disorder Due to a General Medical Condition (stroke), with delusions (293.81). On Axis II, Mr. A's was diagnosed with Mild Mental Retardation. Stroke and rheumatic fever were listed as Axis III diagnoses. Psychosocial and environmental difficulties involving the primary support group (discord with family members), economic problems (insufficient welfare support) and health issues (imminent cardiothoracic surgery for cardiac valve replacement) were listed on Axis IV as relevant stressors. Axis V (Global Assessment of Functioning) score was 30 (indicating behavior that is considerably influenced by delusions or hallucinations OR serious impairment in communication or judgment).

We have also employed range of tests aimed at identifying underlying cognitive deficits, including a disexecutive syndrome (i.e. with go no-go and pattern completion tests [10]), spatial neglect [with line bisections [11] and face-hand tests [12] and provoked confabulations (i.e. with verbal/visual recognition tasks [13]). Importantly, while our patient did not show signs of a right hemisphere syndrome nor of confabulations, we have noted some evidence regarding the presence of poor inhibitory control on go-non-go test. Of note, no spontaneous confabulations [13] was noted during repeated mental status examination. No significant impairment could be identified on Mr. A's more recent neurological examination, and she was able to function without major problems on motor and sensory realms.

Ms. A was initially treated with quetiapine, with some improvement in the first month of treatment. However, due to financial difficulties, she discontinued the medication by herself and displayed worsening of symptoms after a few weeks. At this time, Ms. A showed significant behavior deterioration, with persecutory delusions that culminated in an attempted murder of her older sister, who was stabbed on her back. Ms. A was hospitalized for nearly two months in another mental institution. There, she was prescribed low dose quetiapine (50 mg/day) and citalopram (20 mg/day) with some improvement. After discharge, she returned to treatment in our outpatient clinic.

Despite showing remission of irritability and aggressiveness, Ms. A was still very suspicious about the family. Therefore, quetiapine was increased to 100 mg daily, this time obtained for free through the Brazilian health system. Dose adjustment was followed by an additional improvement of the delusional symptoms, sleep and mood. Complaints of excessive sedation precluded further adjustments in dose at this time. Nevertheless, after one year of treatment, she became once again suspicious and felt persecuted by a moneylender who she thought have come into his house pretending to be an employee coming to fix her washing machine. Quetiapine dose was then increased to 200 mg daily. Currently, the persecutory delusions are under remission and the patient keeps being regularly followed by our psychiatry clinic.

## 3. Discussions

In this study, we describe the case of a patient who developed a psychotic episode approximately one year after a right middle cerebral artery stroke. We believe this case has several aspects worth noting: (1) the rarity with which psychotic episodes occur in victims of stroke, (2) the long interval between the episode and the appearance of psychotic symptoms, (3) the severity and persistence of the psychotic episode, which culminated in an attempted murder and (4) the association between delusional symptoms and injury to the right fronto-temporo-parietal region.

Although cases of psychiatric disorders taking place after vascular brain injury are frequent, the scenario seems to be different when it comes to psychosis. For instance, in a prospective study performed in 2004, Kumral and Ozturk [7] evaluated 360 stroke patients and found that only 4% of these exhibited some kind of delusional symptom. Thus, based on these later figures, cases such as ours should be considered relatively rare. This is fortunate, since post-stroke psychosis has been associated with poorer prognosis. In the cohort by Almeida and Xiao [14], stroke patients with an incident psychotic disorder were twice as likely to die during the subsequent 10 years as post-stroke controls with no mental disorder.

Moreover, the interval between the vascular episode and the appearance of delusional symptoms was nearly one year, a relatively long period, not commonly described in the literature. For example, in the same case series described above by Kumral and Ozturk [7], delusions always occurred until the third day after the vascular event. Other case reports of post-stroke delusions followed a similar pattern and often had an acute onset [5,15]. There were also cases where psychosis was the first symptom, predicting the existence of a stroke not yet diagnosed [16,17].

There is also another interesting finding related to the atypical mode of onset exhibited by our patient. Despite being rare, delayed psychosis after stroke was already related to the presence of post stroke seizures [18,19]. However, as opposed to previous reports, our patient did not exhibit epilepsy, which was excluded on a clinical basis. In addition, she showed marked improvement while on a drug that can worsen seizure control in epileptic patients (i.e. quetiapine). For instance, in a study using WHO adverse drug reactions database, Kumlien and Lundberg [20] found that quetiapine was among the ten neuroactive drugs most commonly associated with drug-induced seizures.

One might argue that not having a seizure while on quetiapine is not a potent argument against the presence of epilepsy. However, we also believe that the improvement of psychosis on quetiapine does not support an etiological role for an epileptic discharge in our particular patient. Therefore, our case suggests that non-epileptic mechanisms can also give rise to delayed onset psychosis in stroke patients. We suspect that some sort of cortical reorganization may play a role in the origins of delayed-onset post-stroke psychosis [21].

The occurrence of an attempted murder as a consequence of a post-stroke delusional disorder is another noteworthy aspect. Although Angelelli et al. [4] observed that irritability is one of the most common behavioral symptoms in the first year that follows a stroke, case reports of severe aggression are not common in this context. However, the persecutory content of the delusion has been commonly seen in similar cases, such as the ones reported Kumral and Ozturk [7], where 67% of delusions were of persecutory type.

Neuropsychiatric symptoms such as depressed mood, emotional lability, compulsive rituals, and delusions are often attributed to dysfunction of frontal-subcortical circuits relevant to the modulation of cognition, emotion, and behavior [5]. Injuries to fronto-temporo-parietal regions, such as those presented by our patient, can lead to a fronto-subcortical disconnection and ultimately, to a variety of neuropsychiatric symptoms. While most of the reports of poststroke delusional disorders are associated with lesions in the right hemisphere [7,9,16], it has not yet been possible to identify a clear relationship between the hemisphere affected by the stroke and the resulting neuropsychiatric symptoms. We believe that underlying deficits in inhibiting prepotent responses (as evidenced by go non-go test) may contribute to the perpetuation of delusional beliefs. In fact, previous studies already showed that positive symptoms are related to a reduction in cognitive inhibition [22].

Our study has several limitations inherent to a case report. For instance, the association between psychosis and stroke can be random, since both conditions are commonly observed in the general population. However, the fact that the patient has an advanced age for the installation of a primary psychotic disorder and did not show signs of delusional symptoms before the event speaks against this hypothesis. Also, although Ms. A was on citalopram during her psychotic episode, we consider unlikely that this drug had any role in the precipitation of her condition. In fact, citalopram was being regularly prescribed for almost one year, with excellent tolerability. In the Cornell Bipolar cohort, antidepressant-induced mania/hypomania occurred much earlier (i.e. $36 \pm 32$ days after antidepressant initiation) [23].

Although we cannot exclude the possibility of an additional stroke as a source of Ms. A psychotic episode, we believe that the occurrence of a new lesion as a cause of delayed psychosis in the absence of significant neurological deficits is unlikely. In fact, while we have attempted to rescan the patient to check for new and/or additional lesions, she was unwilling to be examined and getting into an MRI tube. Finally, future studies should investigate the role of premorbid cognitive deficits, such as those presented by the patient, as predisposing factors for post-stroke psychosis [17].

## Acknowledgments

This work was supported by grant from CNPq, FAPERJ, and IDOR.
